# Grey voters and populist voting. The role of religiosity and national collective narcissism

**DOI:** 10.1371/journal.pone.0336831

**Published:** 2026-09-10

**Authors:** Agnieszka Turska-Kawa, Irena Pilch

**Affiliations:** 1 Institute of Political Science, University of Silesia, Katowice, Poland; 2 Institute of Psychology, University of Silesia, Katowice, Poland; The University of Warwick, UNITED KINGDOM OF GREAT BRITAIN AND NORTHERN IRELAND

## Abstract

The current study aimed to explore the role of religiosity and the social psychology concept of collective narcissism in political behaviour. Individuals endorsing national collective narcissism perceive their nation as superior to others, which is accompanied by the demand that other individuals or nations accept and confirm this superiority. We focused on the process of supporting a populist party in the segment of the oldest citizens, referred to as grey voters. Our research investigates the determinants of voting behaviors and fits into discussions on aging societies. It is an important issue nowadays because demographic change creates a space of challenges, the significant consequences of which we can observe in the electoral market. Our study results confirmed the relationship between religiosity and populist voting and the mediating role of collective narcissism in this relationship. We also found religious faith to be a stronger predictor of populist voting than religious practice. Our results are discussed taking into account previous studies’ results and the Polish cultural, social, and political context.

## Introduction

In recent years, there has been much discussion on the rise of populism in Central and Eastern Europe [1–5]. However, as populists emerge victorious in one country after another, the results of the Polish parliamentary elections held on October 15, 2023, may suggest a weakening of this political trend. It should be clearly emphasized that it was no landslide change, with the populist Law and Justice (PiS) party, which had been in power for the previous eight years, still receiving the greatest support (35.4%). The democratic parties, however, won the majority of the seats in the Sejm, genuinely giving rise to a departure from the undemocratic measures independently implemented by PiS over recent years.

Our study fits within the discussions exploring the determinants of the change noted. We focus our attention on the voting behaviours of the oldest voter segment, referred to as grey voters. Interest in this group stems from projections concerning ageing populations [6]. This is because demographic change has a significant impact on transformations within electorates, for instance with regard to the structure of needs, expectations towards decision-makers, and political communication during election campaigns. In the 2023 parliamentary election, more than 50% of grey voters voted for the populist Law and Justice party. This result shows that the oldest voters are not homogeneous in this respect after all, so it will be interesting to look at the potential drivers of their decisions, i.e., the factors encouraging them to support the populist party.

Research shows that the support for populist parties in Europe is generally stronger among the older generation [2,7]. Importantly, the period of socialization of Polish grey voters occurred at a time when national identity was reviving in the church environment [8]. The Church acted as the defender of the nation and as democratic opposition back then. This was taking place during a period of development that was extremely sensitive to the formation of the respondents’ social and political attitudes. Researchers have been fairly consistent in emphasizing the importance of early political experiences for the development of political attitudes and behaviours later in life [9–12]. The intertwining of these factors allows us to focus on the role of **religiosity** and national **collective narcissism** (CN) in the decision-making process when choosing to support the populist party.

Religions strongly emphasize the distinction between good and evil which is in line with the moral dichotomy present in populism between the good people and the evil elite. Some researchers even indicate the quasi-religious connotations of populism, defining ‘populism as a political style that sets “sacred” people against two enemies: “elites” and “others” [13]. An Italian study has shown that religion can be used in various ways by populist parties [14]. It can be used as an ‘identity marker’ that is highly salient and an instrument for framing specific topics, or as an identifier, present but less salient and used to frame citizenship in juridical/legalist terms. Interestingly, research [15] shows that, despite frequent narrative references to religion made by populist parties, these references are almost absent from their official political programs. According to the authors, this may indicate that populist political parties frame religion for strategic and societal reasons.

In our research, we took into account two forms of religiosity: religious faith, understood as deeply lived religious belief, and religious practice, i.e., the behavioural aspect of religiosity [16], measured in our study as Mass attendance. In turn, collective narcissism, a construct from the field of social psychology, is a form of narcissism related to a reference group an individual identifies with. That group is perceived as superior to others, which is accompanied by the demand that other individuals or groups accept and confirm this superiority [17]. Collective narcissism has recently been studied in the context of various political attitudes and behaviours [18–21]. In our study, we focused on national collective narcissism (CN), whose elements we find in populist rhetoric. CN is defined as a ‘belief that the nation’s exaggerated greatness is not sufficiently recognized by others’ [22].

### Cultural background

According to Herbert [23], ‘the Polish language and the Catholic Church became the twin pillars of national identity’, firmly rooted in Poland’s history. The fusion of religious and national identities is a product of favourably homogenous demographics and historical political opportunities. This relies on the notion that religion stands on the same side as the nation, that when oppression threatened the country in the past, religion protected national representatives and safeguarded national identity [24]. Populist parties can exploit the political potential of Polish religious identity.

The Polish national identity also contains elements compatible with collective narcissism (CN). The national narrative and historical memory place great emphasis on difficult periods for the country, strongly rooted in historical events such as the partitions of Poland and territorial aggression. More than half of all Poles claim that we have suffered just like other nations. However, as many as 43 percent are convinced that we suffered more, which may indicate a strong belief in the uniqueness of one’s own suffering (report: Polskość w XXI wieku. Rodzaje identyfikacji narodowej, ich podłoże i konsekwencje [Polishness in the 21^st^ century. Types of national identification, their background and consequences]). Historical events and the trauma that lingers and is publicly nurtured generate, among other things, hysterical reactions to statements undermining Poland’s status solely as a victim, or distrust of other nations [25].

### Current study: Religiosity, collective narcissism, and voting for populist parties

Research on the relationships between religiosity and voting for populist parties has produced inconsistent results. The discussion concerns the role of the Christian religion as a factor preventing or encouraging voting for radical right-wing populist parties. Marcinkiewicz and Dassonneville [26] found no association or a negative association between religiosity operationalized as church attendance and populist voting in Western Europe but obtained opposite findings for Eastern European countries: Poland and Hungary. Similar results have also been found in other studies [27–29] Positive associations of religiosity with populist voting in various Western European countries, however, have been described [30] in relation to adherence to religious norms. These differences have been explained by different operationalization of variables, cultural context and other contextual variables [31,32]. Although findings from previous research on the relationship between religiosity and populist voting are inconclusive, we can assume that for older people, for whom religious values are an integral part of identity, religiosity will be a driver when it comes to voting for populist parties. This is particularly the case if these parties (such as Law and Justice) become guarantors for the protection of the interests of believers and of the Catholic Church, and advocate Catholic values in the public discourse, dissociating themselves from a secularizing Europe. For this reason, we hypothesized a relationship between religiosity and populist voting.

**Hypothesis 1a**. *Religiosity will be a significant positive predictor of populist voting,*

Research into CN, in turn, clearly shows that it can predict voting for populist parties [19–21,33]. In Hungarian research, collective narcissism was a significant predictor of support for the ruling populist party [20]. In another study, national collective narcissism predicted support for the populist party in Poland and accounted for the relationship between group relative deprivation and support for Donald Trump [21].

**Hypothesis 1b.** *CN will be a significant positive predictor of populist voting.*

Research shows that different forms of religiosity are associated with religious CN, i.e., a form of CN in which the reference group is a religious group [34–36]. In turn, strong associations between national CN and religiosity have been confirmed in studies on representative groups of Poles [37]. Importantly, religious CN and national CN have also been shown to be strongly correlated in Poland [37–39], which further confirms the strong links between CN and religiosity among Poles. On the basis of that research, we put forward Hypothesis 2 on the relationship between religiosity and CN.

**Hypothesis 2.** *Religiosity will be positively associated with CN.*

At the same time, we propose CN as a potential mediator of the relationship between religiosity and populist voting (Hypothesis 3). We predict that religiosity may intensify CN, which in turn may induce people to vote for a party promoting ideas aligned with their own perception of the Polish nation and seeking to restore the latter’s rightful place among other nations. When religious and national themes become intertwined within national identity, supplemented by beliefs about the uniqueness and special role of one’s own nation in the context of religious allegiance and the defence of values associated with it faced with a secularized Europe, religious individuals are the ones who can more easily build and reinforce a system of beliefs building CN. By practising religion, religious people have the opportunity to more frequently evoke and perpetuate elements of identity constituting manifestations of CN, which in turn can reinforce and intensify these attitudes, as well as the related political behaviours, including voting behaviour. CN can induce individuals to vote for a party whose communication not only highlights traditional values and beliefs that are important to them but also openly preaches the greatness and moral superiority of the Nation, at the same time calling itself the sole representative of that Nation and promising to ‘fight’ for the respect due to it. Such communication can satisfy individuals’ narcissistic needs and encourage them to vote for the respective party. In summary, when personal religiosity is linked to a narcissistic need to constantly confirm the superiority of one’s own reference group, as it happens in Poland [37], it may potentially have an impact on voting behaviour via CN.

**Hypothesis 3.** *CN will significantly mediate the relationship between religiosity and populist voting.*

## Materials and Methods

### Participants and procedure

The study involved 379 people aged 62–95, with a mean age of 72.5, 234 women and 145 men, of whom 125 respondents declared to be single (103 widowed, 22 divorced), 224 married, and 30 in informal relationships. Education: 260 respondents declared 12 or fewer years of education, while 119 respondents declared more than 12 years. Of the 379 respondents, 341 stated they participated in the 2023 election to the Polish Sejm, while the remainder declared no participation (33 respondents) or gave no answer (5 respondents). Of the 341 people declaring participation in the election, one person had cast an invalid vote, 39 persons refused to answer, and 301 persons indicated the party they had voted for, including 107 indications of the populist party (PiS). Some people refused to answer questions on religiosity (religious faith 25 persons, religious practice 29 persons). Cases with missing data were excluded from relevant analyses.

The study is a part of a larger project devoted to research on the determinants of voting behaviour of older people (‘grey voters’). A Poland-wide random sample of older people (65 and over, N = 2,082) was sampled from the PESEL (the national identification number) register, taking into account the territorial division of Poland (16 provinces). The researchers attempted to contact each individual drawn from the population and to obtain their consent for an interview. The first wave was conducted on 455 people, and the second wave on 379 people, and it was during the latter that the data used in this study were obtained. The CAPI (Computer Assisted Personal Interviewing) method was used. The research was conducted by a team of trained interviewers, selected in a Poland-wide competition among junior researchers and PhD candidates in the field of social sciences. The study was conducted between October 16 and November 30, 2023.

This study was approved by the Ethics Committee of the University of Silesia (KEUS277/09.2022). Written informed consent was obtained from the respondents before the research. The study was performed according to the Declaration of Helsinki. The dataset for the current study is freely available on the repository: https://doi.org/10.5281/zenodo.18114415. This research was funded by the National Science Centre, Poland (grant no 2021/41/B/HS5/00102) awarded to the first author.

### Measures

**Religious faith.** This aspect of religiosity was measured with the Polish version of the brief version of the Santa Clara Strength of Religious Faith Questionnaire [SCSORF 40,41]. It consists of five items with the answers on a four-point scale (1 – strongly disagree, 4 – strongly agree). To compute overall scores, the items were averaged. Consequently, the overall score retained the original 1-to-4 scale format. Sample item: ‘My religious faith is extremely important to me.’ The measure’s reliability in our study was very good (Cronbach’s alpha = .91).

**Religious practice.** The behavioural aspect of religiosity was measured with a one-item measure. Participants answered the question ‘How often do you participate in Mass and services (also via radio, television, or the Internet)?’ choosing one of the following answers: 1 – never; 2 – once to several times a year; 3–1–3 times a month; 4 – once a week; 5 – more than once a week. For the analysis, the variable was dichotomized: those who declared not practising or doing so occasionally (not more than a few times a year – answers 1 and 2) were included in group 1 (N = 117), while the others, regularly practising at least once a month, were included in group 2 (N = 233).

**Collective (national) narcissism** was assessed with the Polish version of the Short Collective Narcissism Scale [42]. It consists of 5 items with the answers on a six-point scale (1 – strongly disagree, 6 – strongly agree). To compute overall scores, the items were averaged. Consequently, the overall score retained the original 1-to-6 scale format. Sample item: ‘The Polish nation deserves special treatment.’ The reliability of the measure in our study was very good (Cronbach’s alpha = .90)

**Populist voting.** Voting behaviour was assessed by two items: (1) ‘Did you vote in the 2023 parliamentary election?’ (yes/no), (2) ‘If yes, which party did you vote for?’ Voting for PiS was considered a populist vote, and voting for any other party was a non-populist vote. There is consensus among scholars that Law and Justice (PiS) is a populist party [e.g., 43,44]. PiS was also identified as a populist party on the PopuList [45].

### Statistical analyses

Statistical procedures were performed using IBM SPSS Statistics version 26. For all analyses, the significance level was set at *α* = .05. Preliminary analyses were conducted to compute summary descriptive statistics, including means (*M*) and standard deviations (*SD*) for continuous variables (collective narcissism, religious faith and age). Additionally, Pearson’s correlation coefficients were calculated to examine the preliminary bivariate relationships between all study variables. For relationships involving dichotomous variables, this approach functions as a point-biserial correlation. Dichotomous variables were dummy-coded (populist voting 1 = yes, 0 = no; religious practice: never or very rarely = 1, systematically = 2; gender 1 = women, 2 = men; education 12 years or less = 0, more than 12 years = 1), meaning that a positive correlation coefficient indicates higher scores on the continuous variable for the category assigned the higher numerical code.

To test **Hypothesis 1,** multivariable binary logistic regression was used, which is considered a standard approach for binary classification [46]. Our dependent variable (i.e., populist voting) was dichotomous (1 when the participants voted for the populist party and 0 when they voted for the other party), two predictors (religious faith and CN) were continuous variables and one predictor (religious practice) was dichotomous. The controls used were sociodemographic variables: age, gender, and education. As two indicators of religiosity were highly correlated (r = .57, p < .001), and such multicollinearity can result in imprecise results of logistic regression [47], two separate regressions were run. All the variables were entered into the regression in a single step. The bootstrap method with 1,000 bootstrap samples and percentile confidence intervals was used.

Prior to interpreting the models, four core statistical assumptions were tested. Multicollinearity among predictors was assessed using Variance Inflation Factors (VIF); all VIF values for both models were well below the conservative threshold of 2.5 (range 1.01–1.26), indicating no multicollinearity issues. The assumption of linearity between the continuous independent variables and the logit of the dependent variable was verified and confirmed using the Box-Tidwell transformation test, where all interaction terms (X*ln(X)) were statistically non-significant (p > .05). Independence of observations was guaranteed by the cross-sectional research design. Residual diagnostics, including Cook’s distance and standardized Pearson residuals, revealed no influential outliers or anomalous data points affecting the model stability. Model fit was evaluated using the Hosmer-Lemeshow goodness-of-fit test and Nagelkerke’s Pseudo-R^2^.

**Hypothesis 2** concerning the direct relationships between religiosity (religious faith and religious practice) and CN was verified by correlation analysis. To examine these relationships, Pearson correlation analyses were first conducted. To rule out the potential confounding effects of sociodemographic variables (age, gender, and education), partial correlation analyses were subsequently performed.

To test **Hypothesis 3**, namely that CN mediates the effects of religiosity on populist voting, bootstrapped logistic regression with Huber-White robust standard errors was performed. The SPSS Process Macro (Model 4) was used [48]. Two separate models were run with religious faith and religious practice as independent variables.

The mediation model is specified by two regression equations. First, the mediator (M, collective narcissism) is modeled using ordinary least squares (OLS) regression:


$$\begin{array}{c} M=i_{M}+aX+c_{\text{1}\text{1}}K_{\text{1}}+c_{\text{1}\text{2}}K_{\text{2}}+c_{\text{1}\text{3}}K_{\text{3}}+e_{M} \end{array}$$

where *i*_*M*_ represents the intercept, *a* is the coefficient representing the effect of religiosity on CN, c_11_ – c_13_ are the coefficients for the covariates (age, gender and education), and *e*_*M*_ is the residual error term.

Second, the binary outcome (Y; populist voting) is modeled using logistic regression: $\text{logit}(P(Y=\text{1}))=i_{Y}+c^{\prime }X+bM+c_{\text{2}\text{1}}K_{\text{1}}+c_{\text{2}\text{2}}K_{\text{2}}+c_{\text{2}\text{3}}K_{\text{3}}$

where *Y* denotes populist voting (0 = non-populist vote, 1 = populist vote) and logit(P(Y = 1)) represents the log-odds of voting for a populist party, i_Y_ is the intercept, *c’* represents the direct effect of religiosity on populist voting while controlling for CN and the covariates, *b* represents the effect of CN on the log-odds of populist voting after adjusting for religiosity and the covariates, and *c*_21_ *– c*_23_ represent the effects of the covariates in the outcome model.

The indirect effect of religiosity on populist voting via CN is calculated as the product of the coefficients (*a* x *b*). It was estimated using 5,000 bootstrap samples to obtain 95% percentile confidence intervals. Regression coefficients are reported as unstandardized coefficients (*B*), with logistic regression coefficients expressed on the log-odds scale.

Statistical assumptions (including multicollinearity and linearity of the logit) were comprehensively verified during the primary logistic regression stage. Given that the mediation model involved the same predictors, re-testing of these assumptions was deemed redundant and was not performed.

The sample size was not determined a priori. However, in the present study, the number of events per predictor variable (EPV) was 21.4 (107 events/5 predictors) for both the multivariable logistic regression models and for the mediation analyses. This value substantially exceeded the recommended minimum of 10 events per predictor [49], indicating adequate stability of the regression estimates.

In addition, *post hoc* power analyses were conducted using G*Power 3.1 to evaluate whether the obtained sample size was sufficient to test the study hypotheses. For the multivariable logistic regression analyses (Hypothesis 1), the achieved statistical power exceeded .99 for CN (Models 1 and 2), was .999 for religious faith (Model 1), and .80 for religious practice (Model 2). Thus, the obtained sample size provided sufficient statistical power for testing all key predictors according to the conventional criterion of power (≥ .80).

For the correlation analyses (Hypothesis 2), the achieved statistical power was .99 for the association between religious faith and CN and .85 for the association between religious practice and CN, indicating sufficient statistical power for both analyses.

For the mediation analysis (Hypothesis 3), conventional post hoc power analysis is of limited value because the sampling distribution of the indirect effect is asymmetric. Therefore, the adequacy of the sample size was evaluated with reference to the simulation-based recommendations of Fritz and MacKinnon [50], which are specifically designed for mediation models. Given the obtained sample size (N = 276), the magnitude of the observed indirect effect, and the statistically significant bootstrap confidence interval excluding zero, the mediation analysis was considered adequately powered to detect an indirect effect of practical significance.

Taken together, the EPV criteria, *post hoc* power analyses, and the statistically significant bootstrap-mediated effect all suggest that the obtained sample size was adequate for testing the proposed hypotheses.

## Results

Correlations between the variables and descriptive statistics for continuous variables are displayed in Table 1.

**Table 1:** Means, standard deviations, and correlations between study variables.

Variable	Collective narcissism	Religious faith	Age	M	SD
CN	–	–	–	3.89	1.28
Religious faith	.35***	–	–	2.71	0.83
Age	.02	.10	–	72.55	6.22
Religious practice	.18***	.57***	.10	–	–
Gender	−.07	−.08	−.03	–	–
Education	−34***	−.15**	.04	–	–
Populist voting	.52***	.40***	−.02	–	–

The point biserial correlation was calculated for relationships between dichotomous variables (gender, education, religious practice, and populist voting) and continuous variables. M – mean; SD – standard deviation.** p < .01; *** p < .001.

The results of multivariable logistic regression analyses are presented in Table 2.

**Table 2:** Results of multivariate regression analysis.

Predictor	B	Bootstrap 95% CI B	Bootstrap p	OR	95% CI OR
Model 1					
Religious faith	0.93	0.54; 1.51	< .001	2.54	1.68; 3.82
CN	1.09	0.77; 1.53	< .001	2.97	2.13; 4.16
Age	−0.02	−0.08; 0.02	.333	0.97	0.93; 1.03
Gender	0.57	−0.06; 1.29	.076	1.77	0.94; 3.33
Education	0.59	−0.11; 1.41	.120	1.80	0.87; 3.70
Constant	−6.21	−10.61; −2.52	.002	0.01	–
Model 2					
Religious practice	1.24	0.50; 1.99	.002	3.47	1.73; 6.93
CN	1.21	0.93; 1.66	< .001	3.34	2.40; 4.65
Age	−0.02	−0.07; 0.03	.387	0.98	0.93; 1.03
Gender	0.54	−0.08; 1.26	.102	1.71	0.92; 3.19
Education	0.61	−0.08; 1.37	.076	1.84	0.91; 3.71
Constant	−5.21	−9.35; −1.62	.006	0.002	–

B – unstandardized regression coefficient. *OR* – odds ratio. CI – confidence intervals. Variable coding: populist voting (1 = yes, 0 = no); religious practice (1 = never or very rarely, 2 = systematically), gender (1 = women, 2 = men), education (0 = 12 years or less, 1 = more than 12 years).

**Model 1** included religious faith, CN, and demographic controls. The analysis shows a significant relationship between the focal predictors and populist voting. Both CN and religious faith have significant positive effects on voting for a populist party. Age, gender, and education have no impact on vote choice. Hosmer and Lemeshow’s test was insignificant (chi^2^ = 6.02, df = 8, p = .64), suggesting that the model fit the data. Nagelkerke’s pseudo-R^2^ equals 0.44.

**Model 2** included religious practice, CN, and demographic controls. Again, the analysis shows a significant relationship between the predictors and populist voting. CN and religiosity have significant positive effects on voting for a populist party. Age, gender, and education have no impact on vote choice. Hosmer and Lemeshow’s test was insignificant (chi^2^ = 3.52, df = 8, p = .90), and Nagelkerke’s pseudo-R^2^ equals 0.41.

Overall, our data supports Hypothesis 1, namely that religiosity and CN are significant and unique predictors of populist voting. The values of Nagelkerke’s R^2^ suggest that the model with religious faith can predict populist voting slightly better in comparison with the model that includes religious practice.

Consistent with Hypothesis 2, the results revealed significant positive correlations between CN and both dimensions of religiosity (*r* = .35, *p* < .001 for religious faith and *r* = .18, *p* < .001 for religious practice). After controlling for age, gender, and education, both relationships remained statistically significant (*r*p = .32, *p* < .001 for religious faith and *r*p = .14, *p* = .007 for religious practice), indicating that these associations were independent of the controlled covariates. Furthermore, the correlation between religious faith and CN was significantly stronger than the correlation between religious practice and CN (*z* = 3.76, *p* < .001).

**Fig 1:**
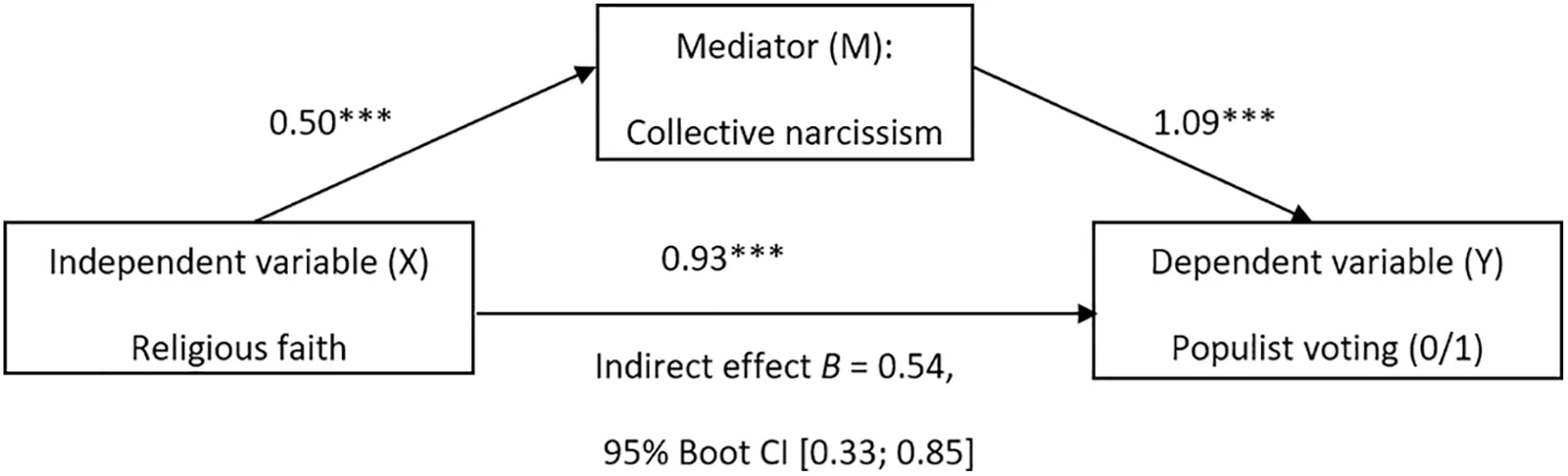
Mediation Model A for populist voting. Path *a* represents the effect of religious faith on CN. Path *b* represents the effect of CN on populist voting. Path *c′* represents the direct effect of religious faith on populist voting. Coefficients on paths *b* and *c*′ are expressed as Log-Odds (*B*). Age, gender, and education were included as covariates in both the mediator and outcome models but are omitted from the figure for clarity. ** *p* < .01; *** *p* < .001.

**Fig 2:**
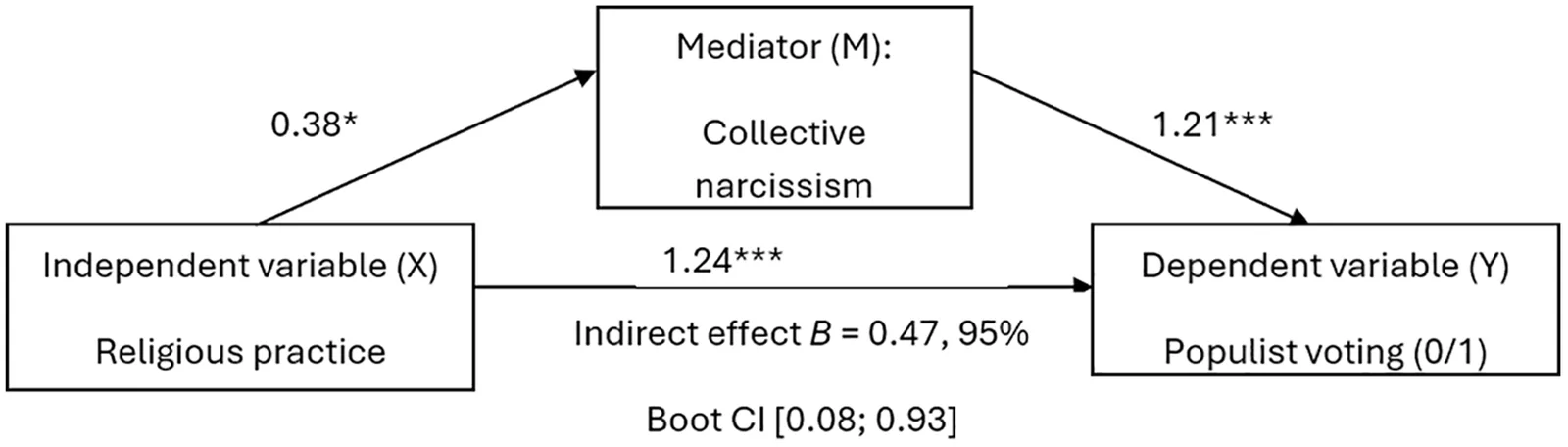
Mediation Model B for populist voting. Path *a* represents the effect of religious practice on CN. Path *b* represents the effect of CN on populist voting. Path *c′* represents the direct effect of religious faith on populist voting. Coefficients on paths *b* and *c′* are expressed as Log-Odds (*B*). Age, gender, and education were included as covariates in both the mediator and outcome models but are omitted from the figure for clarity. * *p* < .05; ** *p* < .01; *** *p* < .001.

The mediation analyses (Table 3, Figs 1 and 2) indicate that CN mediates the relationship between religiosity and populist voting in both Model A (religious faith) and Model B (religious practice).

**Table 3:** Results of mediation analyses.

Predictors	Outcome variable							
		M (CN)				Y (populist voting)		
		B	95% CI	p		*B*	95% CI	*p*
Model A								
X (religious faith)	a	0.50	0.33; 0.67	< .001	c’	0.93	0.52; 1.34	< .001
M (CN)		–	–	–	b	1.09	0.75; 1.42	< .001
constant		2.88	1.17; 4.58	.001		−6.79	−11.00; −2.58	.002
Model B								
X (religious practice)	a	0.38	0.07; 0.69	.02	c’	1.24	0.55; 1.94	< .001
M (CN)		–	–	–	b	1.21	0.88; 1.54	< .001
constant		3.40	1.63; 5.18	< .001		−6.99	−11.16; −2.82	.001

B – unstandardized regression coefficient. X – independent variable. M – mediator variable. Y – dependent variable. CI – bootstrap confidence intervals.

For **Model A**, the results show that religious faith is directly and indirectly associated with populist voting through its effects on CN. Higher religious faith is associated with higher CN (path a = 0.50, p < .001), and higher CN is associated with a higher probability of voting for a populist party (path b = 1.09, p < .001). The bootstrap confidence interval for the indirect effect (ab = 0.54) is above zero (bootstrap CI_95_ = 0.33; 0.85), which means that the indirect effect is significant. Religious faith remains a significant predictor of populist voting after controlling for CN (path c’ = 0.93, p < .001), thus CN only partially mediates this relationship. Nagelkerke’s pseudo-R^2^ equals 0.44.

For **Model B**, the results show that religious practice is directly and indirectly associated with populist voting through its effects on CN. Higher religious practice is associated with higher CN (path a = 0,38, p = .02), and higher CN is associated with a higher probability of populist voting (path b = 1.21, p < .001). The bootstrap confidence interval for the indirect effect (ab = 0.47) is above zero (bootstrap CI_95_ = 0.08; 0.93), thus the indirect effect is significant. Religious practice remains a significant predictor of populist voting after controlling for CN (path c’ = 1.24, p < .001), which indicates that CN partially mediates this relationship. Nagelkerke’s pseudo-R^2^ equals 0.41. Overall, the results support Hypothesis 3.

## Discussion

The aim of the study was to determine the role of religiosity and CN in the decision-making process when choosing to support a populist party in the segment including the oldest citizens, referred to as grey voters. Our research not only investigates the determinants of voting behaviours but also fits into discussions on ageing societies. In fact, demographic change is creating a space of challenges, the significant consequences of which we can also observe on the electoral market. In the course of the research procedure, we put forward three research hypotheses.

The first hypothesis explored religiosity and CN as predictors of populist voting. The study confirmed our predictions, suggesting at the same time that the model with religious faith can predict populist voting slightly better in comparison with the model that includes religious practice. This result is consistent with political science analyses concerning the reasons for the success of the populist party in Poland [e.g., 26,51,52]. The rhetoric proposed by the populist party in Poland gave priority to two issues in its narrative: the threat to Poland’s national interest and position in Europe as well as the defence of Catholic values. In the first case, PiS referred, among other things, to Poland’s history and culture, seeing them as threatened in a secularizing Europe. In the second case, the party made itself into a guarantor of the protection of the interests of believers and of the Catholic Church, for instance by allowing the hierarchs of the Church to participate in the discourse on important political and social issues.

Given the characteristics of the survey respondents, it is worth mentioning in this context the sociological (albeit contested by some researchers) division into ‘Poland A’ and ‘Poland B’. Those who declare to have incurred a loss, among other things, as a result of the political system transformation are more often older people, those living in small towns and villages, those of lower educational attainment, those in the lower income quartile, the unemployed, the retired, and those receiving invalidity benefits: such are the people that have, at least in relative terms, lost out on the advantages coming from the transition [53]. These individuals will therefore naturally be opposed to the opening up of Poland and they will also be more attached to Polish national values. Inhabitants of ‘Poland B’ were characterized by a collectivist and Church-centric conception of their religious beliefs [54]. In the 2015 parliamentary elections, PiS clearly won in the eastern provinces, belonging to ‘Poland B’, which points to a similar direction of the associations of the variables used in our study with voting for the populist party.

The second hypothesis concerned the direct relationships between religiosity and CN. A positive relationship was confirmed for both religious practice and religious faith, the relationship being significantly stronger in the latter case. This result is consistent with other studies on the Polish population [37]. At the same time, this finding confirms the specific nature of Polish national identity, in which the two factors are intertwined. Religiosity in Poland seems to reflect a need to belong to groups sharing a belief in their superiority and in the right to privileged treatment [37]. In countries with one dominant religion, religiosity often co-occurs with higher self-esteem [55] and a tendency to consider oneself better than others [56]. This effect is certainly reinforced by a national narrative concerning historical events, based on trauma and a sense of being wronged. The Church was an active actor in these events, which over time gave it the role of a symbolic protector of the Polish nation. This was particularly evident during the period of the struggle against communism and in the process of transition to democracy, of which grey voters were observers and in which they participated. The nature of the Church’s involvement in transition processes also made it an important actor for non-religious people. It would become a political entity with the approval of the general public, based on the feeling of peace and security provided within it in difficult times [52].

Our study results also confirmed the third hypothesis on the mediating role of CN in the relationship between religiosity and populist voting. Both religious faith and religious practice are directly and indirectly associated with populist voting through their effects on CN. This finding confirmed our conjecture that individual religiosity linked to the need to confirm one’s own group’s superiority was significantly related to voting behaviour via CN. However, our study has a cross-sectional design, thus causal inference is not possible. It therefore remains an open question whether the interaction between religiosity and CN is possible and by what means. In a study conducted among members of Islamic religious groups [36], religious CN was a mediator of the relationship between religious fundamentalism and endorsing extreme group behaviour. In turn, in the study by Federico et al. [37], the longitudinal analysis did not identify cross-lagged effects of religiosity on CN and vice versa. As it is not possible to draw conclusions about causality on the basis of a cross-sectional study, further experimental research is needed to confirm the possibility of CN developing on the basis of religiosity.

In the process of verifying the hypotheses, we found religious faith to be a stronger predictor of populist voting than religious practice. This may reflect the secularization trends that have been observed over the last few years, which, however, have been progressing very slowly, with the respective initiatives usually being short-lived. For example, emerging parties and social movements based on criticism of religious values (e.g., Palikot”s Movement, Robert Biedron’s Spring) would initially gain support, only to disappear from the public space rather quickly [57]. Poland is a country with a population of 37.7 million and 32.19 million registered members of the Roman Catholic Church (Religious Denominations in Poland 2019–2021, 2022). However, due to various factors, we have observed a decline in the number of faithful, with a decrease of 1.51 million since 2011 [58–61]. In Poland, there has been a slow decline in declarations of faith (from 84% in 1997 to 78% in 2024) and an even faster decline in practices (from 50% in 1997 to 34% in 2024), which may partly be responsible for the above difference in predictor strength. Another reason may be that religious faith is more strongly linked to needs (e.g., related to a sense of security, understanding the world), to which populist communication refers. In the context of the differences noted, it is also worth emphasizing that religious practices are to a large extent an expression of faith, constituting one of its components. Religious faith accommodates cognitive, emotional and behavioural elements, which may give it greater predictive power.

The survey was conducted in Poland, which provides a good context for our research. On the one hand, this uniqueness is provided by historically difficult events that continue to be ossified in the public space, and evoked during political elections. On the other hand, there is the religious identity that intertwines with these events and gives them context. Thirdly and finally, Poland is a state that has experienced rapid democratic backsliding amid a rise in populist nationalism [62]. Between 2015 and 2023, the nation was ruled by Law and Justice, a far-right party with an authoritarian-populist orientation that has made nationalism [63] and national collective narcissism [19,22] central to its electoral appeals and its style of governance. This state of affairs was overcome in the 2023 general election and our study fits within the discussions exploring the determinants of the change.

The current study was designed to contribute to the knowledge of the potential determinants of populist voting, which is particularly relevant as populist movements grow in strength and gain increasing interest and support from voters. The results can add to the discussion on the role of religiosity in the political choices of voters and the processes behind it. The results can also expand our understanding of the potential role of a social psychology concept of CN in analysing political behaviour. The survey involved the oldest segment of voters. Despite the increasingly intensive discussion concerning ageing populations, little research has so far been undertaken on the consequences of these processes for the electoral market. Our study contributes to this discussion by exploring the specific nature of grey voters’ voting behaviours.

While our sample was entirely random, it was not gender-balanced, as women accepted the invitation to the survey more often. On the other hand, this selection made it possible to include a group of grey voters in our survey comprising people residing in each of the provinces, with different education, status or family situation. This sample is more diverse compared to those recruited using convenience sampling. In addition, the very method used to conduct the survey, assisted by an interviewer whose task was to ensure the interviewees’ comfort and to help persons with disabilities, is an advantage of this survey.
